# A Composite Layered Piezoelectric Pressure Sensor for Dynamic Monitoring with Enhanced Sensitivity and Temperature Adaptability

**DOI:** 10.3390/s26134202

**Published:** 2026-07-03

**Authors:** Suyue Liu, Dazhao Zhou, Jinghua Lin, Jifang Tao

**Affiliations:** 1 School of Information Science and Engineering, Shandong University, Qingdao 266237, China; yuesu@mail.sdu.edu.cn (S.L.); lyn.lin@mail.sdu.edu.cn (J.L.); 2 Key Laboratory of Laser and Infrared System of Ministry of Education, Shandong University, Qingdao 266237, China; zhoudazhao6908@mail.sdu.edu.cn

**Keywords:** piezoelectric pressure sensor, composite layered structure, structural optimization, dynamic pressure monitoring, temperature stability

## Abstract

Piezoelectric pressure sensors for dynamic monitoring face a trade-off between charge output and measurement range, and existing high-sensitivity designs are largely confined to narrow ranges. This study presents a composite layered piezoelectric pressure sensor in which a 316L stainless-steel diaphragm drives a centrally suspended PZT-5H wafer supported by a perforated alumina gasket, with the wafer thickness and cavity radius optimized under a 10 MPa full-scale stress constraint. Over 0–10 MPa, quasi-static calibration gave a highly repeatable quadratic pressure–charge relationship ($R^{2}=0.99995$) with a maximum residual below 1% FS. The sensitivity is pressure-dependent: the secant sensitivity increased monotonically from 3.16 pC/kPa at 1 MPa to 5.36 pC/kPa at 10 MPa, reflecting a stress-stiffening response rather than a measurement tolerance band. The output deviation remained within 3% from 25 °C to 150 °C. Shock-tube testing yielded a resonance of ∼50 kHz and a mutually consistent 10–90% leading-edge interval of 10.12 μs. Combining high charge sensitivity over a wide 0–10 MPa range with a fast transient response and stable operation up to 150 °C, the proposed sensor is suited to dynamic pressure-pulsation monitoring in fluid-power and thermal and power-plant fluid systems.

## 1. Introduction

Pressure sensors serve as indispensable core sensing components in diverse engineering fields, including industrial automation, automotive electronics, aerospace systems, and biomedical devices [1]. Their measurement accuracy and operational reliability directly underpin the performance of integrated systems. With the rapid advancement of modern industry, there has been a growing demand for pressure sensors with advanced performance specifications, such as extended measurement ranges, high sensitivity, superior temperature stability, and enhanced robustness under harsh environmental conditions [2]. Conventional pressure sensing technologies, including capacitive and resistive sensors, exhibit inherent limitations in meeting these stringent requirements, thus creating an urgent need for innovative sensing solutions [3,4].

Piezoelectric pressure sensors have attracted considerable research interest owing to their unique advantages, including high sensitivity, rapid dynamic response, excellent temperature characteristics, and self-powered operation enabled by the piezoelectric effect [5,6]. This effect is manifested as the generation of surface charges proportional to the applied mechanical stress, thereby realizing the direct conversion of mechanical energy into electrical signals [7,8]. Despite these merits, conventional piezoelectric sensor designs possess critical structural limitations, such as uneven stress distribution across the sensing element, high susceptibility to electromagnetic interference (EMI), and inadequate static pressure measurement capability [9,10,11]. These limitations are particularly prominent in extreme operating environments, leading to compromised reliability and signal stability [12,13].

Recent advances in materials science and Micro-Electro-Mechanical Systems (MEMS) technology have provided new opportunities to address these inherent limitations. Structural optimization has emerged as a pivotal strategy to improve stress distribution, reduce thermal drift, and enhance sensitivity [14,15,16]. In parallel, the development of lead-free piezoelectric ceramics, such as potassium sodium niobate (KNN) and bismuth-modified systems, has been actively pursued to provide environmentally sustainable alternatives with competitive piezoelectric coefficients and elevated Curie temperatures [17,18].

Although cutting-edge research has achieved remarkable breakthroughs in sensitivity, a closer examination reveals a recurring trade-off between sensitivity and measurement range. On the one hand, voltage-mode designs report very high nominal sensitivities but only over narrow ranges. For instance, Liang et al. developed a flexible lead zirconate titanate (PZT) thick-film sensor exhibiting a maximum sensitivity of 813 mV/kPa, but with an effective range limited to approximately 0–55 kPa [19]; Xu et al. reported high-sensitivity LiNbO_3_ single-crystal sensors confined to low force levels (0.01–1.5 N) [20]. Because such voltage-mode values scale with the built-in amplifier gain, they cannot be directly compared with the intrinsic charge sensitivity (in pC/kPa) of charge-mode devices.

On the other hand, directly comparable charge-mode designs reveal the same range–output tension. Dzuba et al. obtained a high charge sensitivity of 4.4 pC/kPa with an AlGaN/GaN MEMS diaphragm, but only over a low dynamic range up to 36 kPa [21]. Conversely, charge-mode devices that do reach the MPa regime exhibit markedly lower charge output: the all-ceramic LTCC/PZT sensor of Dabrowski and Golonka, designed for pressures up to 6 MPa, delivered only about 0.71 pC/kPa [22], whereas the PZT-5A-based sensor of Li et al., despite an exceptionally high resonant frequency of 237 kHz tailored for blast measurement, provided about 1.13 pC/kPa over a 1.2 MPa calibration range [23]. More recent efforts have largely centered on flexible and wearable architectures for low-pressure (kPa-level) physiological and tactile sensing [24,25].

Taken together, these studies indicate that a high charge output has so far been confined either to narrow, kPa-level ranges or to designs that sacrifice charge output in order to attain a wide range or an ultra-high resonant frequency. A clear gap therefore remains for sensors that sustain a high charge output, on the order of several pC/kPa, across a wide 0–10 MPa range. This need is particularly acute in fluid-power condition monitoring, such as the pressure pulsation of hydraulic pumps, valves, and actuators, and in thermal and power-plant fluid systems, such as water-hammer and pressure-wave monitoring in feedwater and steam lines, where sensors must capture weak, medium-to-low-frequency dynamic pressure fluctuations against a wide-range static pressure background (e.g., 0–10 MPa), often at elevated temperatures.

To address the aforementioned challenges and fill the gap in high-sensitivity sensing over a wide measurement range, this study proposes an innovative piezoelectric pressure sensor featuring a composite structural design that integrates a circular thin piezoelectric wafer (PZT-5H) with a perforated ceramic gasket. Through meticulous structural optimization, this design efficiently converts external pressure into maximized bending strain of the piezoelectric wafer while maintaining structural robustness. The proposed sensor attains a charge sensitivity on the order of 5 pC/kPa (load-dependent, 3.16–5.36 pC/kPa) across a broad measurement range of 0 to 10 MPa while operating stably up to 150 °C.

The novelty of this work lies in the structural integration of materials. Specifically, the contributions are threefold: (i) a perforated-ceramic-gasket architecture that creates a centrally suspended, annularly clamped PZT region to maximize bending strain; (ii) a laminated-plate-guided optimization of the wafer thickness and cavity radius under an explicit full-scale (10 MPa) mechanical stress constraint; (iii) the resulting ability to sustain a high charge sensitivity (∼5 pC/kPa) over the full 0–10 MPa range. Unlike conventional flat-clamped or cavity-backed diaphragm piezoelectric sensors, which generally trade sensitivity for measurement range, this configuration deliberately accepts a weak but well-calibrated nonlinearity in exchange for substantially higher charge output over a wide range.

The remainder of this paper is organized as follows. Section 2 presents the sensor design and theoretical modeling, including the structural design principles and the laminated composite-plate model of electromechanical coupling. Section 3 describes the structural optimization and finite element analysis, covering the simulation model, material parameters, parameter optimization, and mechanical safety verification. Section 4 details the experimental system and test design for the quasi-static, temperature, and dynamic characterizations. Section 5 presents and systematically analyzes the quasi-static and dynamic experimental results, evaluating the sensor’s measurement range, sensitivity, environmental adaptability, and transient response, and benchmarks the proposed sensor against representative piezoelectric pressure sensors and commercial transducers reported in the literature. Finally, Section 6 summarizes the main conclusions and outlines potential directions for future optimization.

## 2. Sensor Design and Theoretical Modeling

### 2.1. Sensor Structure

**Figure 1:**
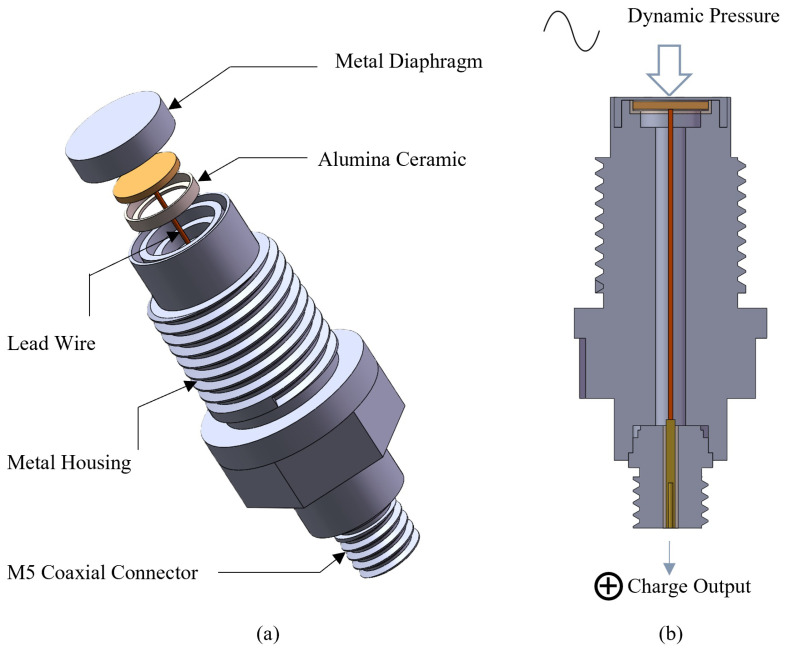
Schematic diagrams of the piezoelectric pressure sensor structure: (**a**) Three-dimensional exploded view with five key components and (**b**) cross-sectional view illustrating the charge generation mechanism under dynamic pressure.

The piezoelectric pressure sensor proposed in this study is a pressure-sensitive element with a composite layered structure. Its core design goal is to enhance sensitivity by optimizing the stress distribution of the piezoelectric wafer, realizing efficient and controlled conversion of external pressure into the bending strain of the piezoelectric wafer via a precision mechanical structure, while ceramic materials are utilized to ensure excellent electrical insulation and mechanical protection without compromising sensitivity. Figure 1a presents a three-dimensional exploded view of the sensor structure, which comprises five key components from top to bottom: a circular metal diaphragm, a circular piezoelectric wafer, a custom ceramic gasket (made of alumina ceramic) with a central hole, a metal housing, and an M5 coaxial connector for exporting charges generated by the sensor. Figure 1b shows a cross-sectional view of the pressure sensor; dynamic pressure signals first deform the top metal diaphragm, and the resulting mechanical deformation is transferred to the PZT-5H piezoelectric wafer (Zhejiang Shenlei Ultrasonic Technology Co., Ltd., Shaoxing, China), generating electric charges that are transmitted through wires to the M5 coaxial connector for low-signal-loss measurement output.

Material selection is fundamental to achieving high sensor sensitivity. The piezoelectric wafer employs a thickness-poled PZT-5H piezoelectric ceramic wafer with dimensions of 5 mm in diameter and 0.5 mm in thickness. PZT-5H exhibits a high piezoelectric constant $d_{31}$ and a high electromechanical coupling coefficient, which are core material properties for realizing high-sensitivity dynamic pressure sensing [26].

Both the metal diaphragm and housing are fabricated from 316L stainless steel (Dongguan Chang’an Ruixiu Precision Hardware Products Factory, Dongguan, China), featuring a specific diaphragm thickness ($h_{m}$) of 0.2 mm. This thickness was selected as a fixed boundary condition rather than an optimization variable, as it represents the lower limit of current precision machining capabilities while effectively reducing the risk of plastic yield failure under the 10 MPa full-scale pressure. The relatively high stiffness of 316L stainless steel enables efficient transmission of pressure-induced deformation to the piezoelectric wafer. It also exhibits excellent corrosion resistance and a thermal expansion coefficient close to that of ceramic materials, facilitating the reduction of thermal stress that may degrade sensitivity stability. The custom ceramic gasket is composed of 99% alumina ceramic (Hangzhou Rechen Special Ceramics Co., Ltd., Hangzhou, China). Owing to its high stiffness and excellent electrical insulation, the alumina gasket provides rigid annular support for the piezoelectric wafer while electrically isolating the lower electrode from the metal housing.

As the primary sensitive and force-bearing element of the sensor, the circular metal diaphragm is rigidly connected to the metal housing via laser welding at its periphery, forming a fixed boundary. This boundary condition ensures that external pressure is concentrated to the central area rather than dispersed, which is crucial for enhancing sensitivity. When external pressure is uniformly applied to the diaphragm surface, the diaphragm undergoes elastic bending deformation and concentrically transmits the pressure to the central area of the underlying piezoelectric wafer in close contact. While the diaphragm thickness was pre-determined by mechanical processing limits, the internal structural parameters were subsequently optimized through finite element analysis (FEA) to balance two key requirements for high sensitivity: no plastic deformation within the rated measurement range, and sufficient driving force for the bending deformation of the piezoelectric wafer to generate large strains.

The custom ceramic gasket is not a simple flat washer but a precision-machined annular component with a special three-dimensional structure. A circular groove with a diameter of 5 mm and a depth of 0.5 mm is machined on the upper surface of the gasket to accommodate the piezoelectric wafer and effectively insulate the side and lower surfaces of the piezoelectric wafer from the metal housing. A through-hole with a diameter of 4 mm is located at the center of the gasket. This configuration forms a centrally suspended piezoelectric region with annular peripheral support, which promotes bending deformation of the wafer under applied pressure. The alumina gasket also serves as a mechanical transition component between the piezoelectric wafer and the metal housing, helping to distribute local contact stress and improve mechanical stability under repeated pressure loading. Its thermal expansion coefficient, relatively close to that of PZT materials, contributes to reducing thermal stress caused by temperature variations, ensuring sensitivity stability.

Both the upper and lower surfaces of the piezoelectric wafer are plated with silver electrode layers to ensure low contact resistance and efficient charge collection. During assembly, approximately 0.5 μL of high-temperature conductive silver epoxy (Zhuhai Jinshineng Technology Co., Ltd., Zhuhai, China) is dispensed between the metal diaphragm and the upper electrode of the PZT-5H wafer to establish a reliable electrical ground connection. This adhesive has an elastic modulus of approximately 3.5 GPa and a rated service temperature of up to 200 °C. Forming an ultra-thin conductive layer (∼25 μm) whose sole function is electrical grounding, it serves no mechanical bonding purpose. The lower surface of the piezoelectric wafer relies purely on physical contact with the alumina gasket for rigid annular support and electrical insulation, without any adhesive.

Synchronous mechanical deformation and interfacial stability are ensured by a static clamping force. The external metal housing is tightened via precision threads using a calibrated torque wrench set to 1.5 N·m, which effectively prevents dynamic interfacial separation under full-scale loads. Furthermore, all core components—including the 316L diaphragm, the alumina gasket, and the PZT-5H piezoelectric wafer—are manufactured and controlled with a strict dimensional tolerance of ±0.05 mm. This matching tolerance helps ensure the coaxial alignment of the internal components. Finally, the lower electrode is connected to an insulated lead wire, which is spot-welded to the central pin of an M5 coaxial connector for low-loss charge extraction.

### 2.2. Theoretical Modeling of Electromechanical Coupling

**Figure 2:**
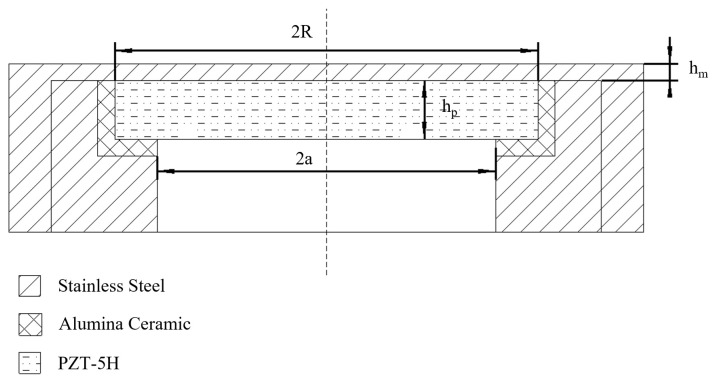
Two-dimensional schematic diagram of the sensor’s core sensing structure.

The core sensing structure and the geometric parameters relevant to the subsequent derivation are illustrated in Figure 2. These parameters, including the diaphragm radius *R*, diaphragm thickness $h_{m}$, piezoelectric wafer thickness $h_{p}$, and ceramic cavity radius *a*, define the overall geometry, while *a*, $h_{m}$, and $h_{p}$ are directly involved in the simplified analytical derivation.

#### 2.2.1. Mechanical Deformation Analysis of the Laminated Composite Plate

When subjected to pressure, the metal diaphragm and the closely contacted PZT-5H wafer are idealized as a laminated composite plate. This analytical formulation assumes idealized contacted interfaces, treating the conductive silver epoxy layer and physical contacts as transferring continuous strain without interfacial slip or compliance. According to classical composite plate theory, integrating the piezoelectric layer alters both the effective bending stiffness and the position of the neutral plane. Setting the coordinate origin z=0 at the bottom surface of the PZT wafer, the equivalent moduli of the metal and PZT layers are defined as $E_{m}^{\prime }=E_{m}/\left(1-\nu_{m}^{2}\right)$ and $E_{p}^{\prime }=E_{p}/\left(1-\nu_{p}^{2}\right)$, respectively. For analytical tractability, the PZT-5H layer is approximated as an equivalent transversely isotropic plate in the radial–circumferential plane. The shifted neutral plane $z_{n}$ of the composite structure is derived as:

(1) $$z_{n}=\frac{E_{p}^{\prime }h_{p}^{2}+E_{m}^{\prime }h_{m}\left(2h_{p}+h_{m}\right)}{2(E_{p}^{\prime }h_{p}+E_{m}^{\prime }h_{m})}$$

Consequently, the effective bending stiffness $D_{eff}$ of the composite plate is obtained by integrating the stiffness over the entire cross-section relative to the neutral plane:

(2) $$D_{eff}=\frac{E_{p}^{\prime }}{3}\left[z_{n}^{3}+{\left(h_{p}-z_{n}\right)}^{3}\right]+\frac{E_{m}^{\prime }}{3}\left[{\left(h_{p}+h_{m}-z_{n}\right)}^{3}-{\left(h_{p}-z_{n}\right)}^{3}\right]$$

Under clamped boundary conditions, the radial deflection distribution w(r) of the composite plate suspended in the central region (radius *a*) under uniformly distributed pressure *p* is determined by:

(3) $$w\left(r\right)=\frac{p}{64D_{eff}}{\left(a^{2}-r^{2}\right)}^{2}$$

Here, the suspended region is idealized as clamped at its edge r=a (the inner rim of the alumina cavity), whereas in the actual device, the diaphragm is welded at the larger housing radius *R* and the gasket provides only near-rigid annular support; this simplification, together with the load-concentrating effect of the surrounding diaphragm, is one source of the residual difference between the analytical and experimental sensitivities discussed below. The analytical model is therefore used for design guidance and geometric scaling rather than as an exact predictor.

#### 2.2.2. Anisotropic Piezoelectric Constitutive Modeling

Addressing the anisotropic characteristics of piezoelectric materials, this model introduces the linear piezoelectric constitutive equations for the PZT-5H wafer. Under the plane stress assumption, the radial strain $\epsilon_{rr}$ and tangential strain $\epsilon_{\theta \theta }$ inside the piezoelectric wafer are linearly distributed along the thickness direction, scaled by the distance from the shifted neutral plane:

(4) $$\epsilon_{rr}\left(r,z\right)=-\left(z-z_{n}\right)\frac{d^{2}w}{dr^{2}},\;\epsilon_{\theta \theta }\left(r,z\right)=-\frac{\left(z-z_{n}\right)}{r}\frac{dw}{dr}$$

To scientifically describe the conversion between strain and stress, this model employs the equivalent plane stress elastic stiffness matrix under a constant electric field. The coupling relationship between the stress field σ and strain field ϵ inside the piezoelectric wafer is expressed as:

(5) $$\left[\begin{array}{c} \sigma_{rr}\\ \sigma_{\theta \theta } \end{array}\right]=\left[\begin{array}{cc} {\bar{c}}_{11}^{E} & {\bar{c}}_{12}^{E}\\ {\bar{c}}_{12}^{E} & {\bar{c}}_{11}^{E} \end{array}\right]\left[\begin{array}{c} \epsilon_{rr}\\ \epsilon_{\theta \theta } \end{array}\right]$$

where ${\bar{c}}_{11}^{E}$ and ${\bar{c}}_{12}^{E}$ represent the equivalent in-plane elastic stiffness coefficients of the piezoelectric ceramic in the radial–circumferential plane. According to the dielectric constitutive relation, the electric displacement component $D_{3}$ in the polarization direction under the influence of mechanical stress coupling is expressed as:

(6) $$D_{3}=d_{31}\left(\sigma_{rr}+\sigma_{\theta \theta }\right)+\epsilon_{33}^{T}E_{3}$$

In a typical short-circuit measurement state, the internal electric field $E_{3}=0$. By substituting the stress–strain relationship, the electric displacement distribution inside the wafer is obtained:

(7) $$D_{3}\left(r,z\right)=d_{31}\left({\bar{c}}_{11}^{E}+{\bar{c}}_{12}^{E}\right)\left(\epsilon_{rr}+\epsilon_{\theta \theta }\right)$$

#### 2.2.3. Quantitative Derivation of Output Charge

The total output charge *Q* is collected at the lower electrode of the PZT wafer (signal electrode), while the upper electrode is grounded. By Gauss’s law, the free charge is governed by the electric displacement at the lower wafer surface (z=0), with lever arm $z_{n}$ relative to the shifted neutral plane. For a clamped circular plate under uniform pressure, the Laplacian $\nabla^{2}w\left(r\right)=\frac{p}{8D_{eff}}\left(2r^{2}-a^{2}\right)$, which sets the in-plane strain sum $\epsilon_{rr}+\epsilon_{\theta \theta }$, changes sign at the curvature-reversal radius $r=a/\sqrt{2}$ [27]. The induced charge is therefore of opposite polarity in the central ($0\leq r<a/\sqrt{2}$) and peripheral ($a/\sqrt{2}<r\leq a$) regions, and a signed integration over the full suspended area vanishes identically. A representative single-polarity contribution can therefore be estimated from the central region; integrating the electric displacement over $0\leq r\leq a/\sqrt{2}$ yields the characteristic charge amplitude:

(8) $$\left|Q\right|=\left|\int_{0}^{a/\sqrt{2}}d_{31}\left({\bar{c}}_{11}^{E}+{\bar{c}}_{12}^{E}\right)z_{n}\nabla^{2}w\left(r\right)\cdot 2\pi r\;dr\right|=\frac{\pi \cdot |d_{31}|\cdot \left({\bar{c}}_{11}^{E}+{\bar{c}}_{12}^{E}\right)\cdot z_{n}\cdot a^{4}}{32\;D_{eff}}\cdot p$$

This closed-form expression contains no adjustable fitting parameter and explicitly relates the output charge to the key structural quantities, namely the composite effective bending stiffness $D_{eff}$, the neutral-plane offset $z_{n}$, and the cavity radius *a*, with a strong geometric dependence $Q\propto a^{4}/D_{eff}$. Equation (8) is therefore used to capture the characteristic charge scale and its parametric dependence on *a*, $z_{n}$, and $D_{eff}$, rather than as an exact predictor of the net terminal charge. For the optimized configuration (a=2.0 mm, $h_{p}=0.5$ mm, $h_{m}=0.2$ mm), substituting the material parameters into Equation (8) yields a characteristic sensitivity estimate of approximately 5.3 pC/kPa, which is consistent in magnitude with the finite-element result (≈5 pC/kPa) and lies within the experimentally measured range (3.16–5.36 pC/kPa). The actual terminal charge is determined by the three-dimensional FEA model, in which the larger welded diaphragm, the annular ceramic support, the packaging constraint, and the non-uniform load-transfer path break the symmetry assumed in the simplified analytical model. The remaining differences between the analytical estimate and the finite-element and experimental values are attributed to the simplifications of the linear small-deflection model, which neglects the load-concentrating effect of the larger diaphragm as well as stress-stiffening and contact effects. Both the analytical and the finite-element models (Section 3) are therefore employed primarily as design-guidance tools; under their idealized contacted-interface assumptions, the predicted response is approximately linear over 0–10 MPa, whereas the weak stress-stiffening nonlinearity near full scale is captured by the experimental characterization in Section 5.

## 3. Structural Optimization and Finite Element Analysis

### 3.1. Finite Element Simulation Model and Material Parameters

To validate the theoretical model and quantitatively determine the optimal structural dimensions, a three-dimensional multi-physics finite element model was established using COMSOL Multiphysics 6.3 (COMSOL AB, Stockholm, Sweden). The simulation fully coupled the Solid Mechanics and Electrostatics modules via the Piezoelectric Effect multiphysics node to capture the direct electromechanical conversion. In the simulation setup, the bottom and outer peripheral surfaces of the metal housing were assigned a Fixed Constraint boundary condition. A uniformly distributed Follower Pressure of 10 MPa was applied to the upper surface of the 316L stainless steel diaphragm. To reflect the idealized contacted interfaces discussed in the analytical model, all physical contact interfaces among the diaphragm, the piezoelectric wafer, and the alumina gasket were configured using the Form Union (Identity) operation, ensuring continuous strain transfer without relative slip. The Ground boundary condition was applied to the upper electrode of the PZT-5H wafer (interfacing with the grounded metal diaphragm), while a Terminal boundary condition (Charge measurement) was assigned to the lower suspended electrode.

Furthermore, the “include geometric nonlinearity” option was activated in the COMSOL Stationary Study to account for potential stress-stiffening under large deformations. As noted in Section 2.2, under the idealized contacted interfaces (Form Union) and the high bending stiffness of the 0.2 mm 316L diaphragm and rigid alumina support, the simulated geometric stress-stiffening over 0–10 MPa is weak, so the simulated response remains approximately linear and underestimates the nonlinearity observed experimentally near full scale; the model therefore serves as an initial-optimization tool rather than an exact large-deflection predictor.

The entire domain was discretized using free tetrahedral elements with quadratic (second-order) Lagrange shape functions, with at least five element layers through the 0.5 mm suspended PZT region and local refinement at the electrode interfaces. A mesh independence study using three successively refined meshes (predefined “Fine”, “Finer”, and “Extra fine” calibrations) is summarized in Table 1; the full-scale (10 MPa) output charge converged, with the consecutive-mesh deviation decreasing from 6.1% to 1.4%. The finest mesh (100,361 elements) was adopted for all subsequent simulations, yielding a converged full-scale sensitivity of approximately 4.96 pC/kPa. In the simulation, the PZT-5H wafer used the built-in COMSOL Material Library data (full anisotropic elastic, piezoelectric, and dielectric matrices), whereas the 316L stainless steel and 99% alumina ceramic were treated as linear elastic materials. The complete material properties are summarized in Table 2.

### 3.2. Parameter Optimization and Mechanical Safety Verification

As analytically derived, the diaphragm thickness $h_{m}$ exerts the most profound impact on sensitivity, since it strongly governs both the composite effective bending stiffness $D_{eff}$ and the neutral-plane offset $z_{n}$. However, $h_{m}$ cannot be indefinitely reduced due to mechanical yield limits under high-pressure impacts. Therefore, $h_{m}$ was configured as a fixed boundary constraint ($h_{m}=0.2$ mm) rather than a sweep variable, representing the lower limit of current precision machining capabilities.

**Figure 3:**
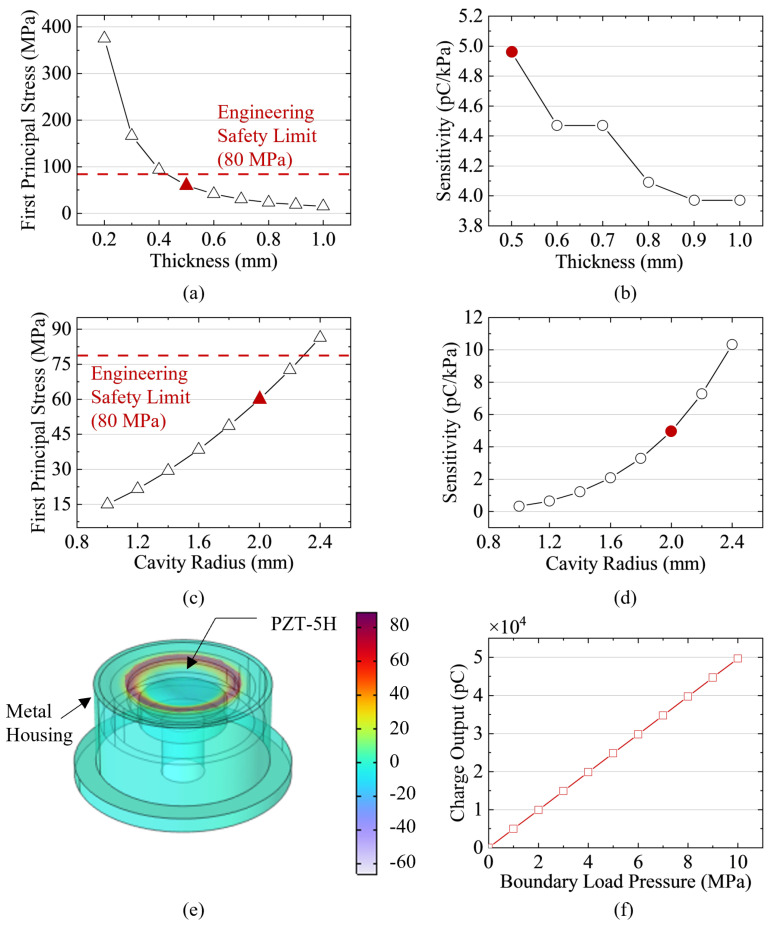
Structural optimization and finite element simulation verification of the composite piezoelectric sensor. (**a**) Effect of piezoelectric wafer thickness ($h_{p}$) on the maximum tensile stress. (**b**) Effect of piezoelectric wafer thickness ($h_{p}$) on the sensing response. (**c**) Effect of ceramic cavity radius (*a*) on the maximum tensile stress. (**d**) Effect of ceramic cavity radius (*a*) on the sensing response. (**e**) Stress distribution contour map of the optimized structure ($h_{p}=0.5$ mm, a=2.0 mm) under a full-scale load of 10 MPa. (**f**) Linear relationship between the output charge and the applied pressure for the final optimized design. The red dashed lines in (**a**) and (**c**) indicate the 80 MPa engineering safety limit of PZT-5H. The optimal design points are highlighted with solid symbols, whereas other data points are represented by hollow symbols.

With the diaphragm thickness fixed, the geometric optimization focused strictly on the internal piezoelectric parameters: the piezoelectric wafer thickness $h_{p}$ and the radius of the suspended cavity *a*. Figure 3a,b illustrate the influence of varying piezoelectric wafer thickness $h_{p}$ on the maximum tensile stress and the sensing response, respectively. Similarly, Figure 3c,d demonstrate the effect of the ceramic cavity radius *a*. As explicitly indicated by the red dashed lines in Figure 3a,c, the maximum tensile stress must be evaluated against the 80 MPa engineering safety limit of PZT-5H. The FEA results capture the underlying physics: within the mechanically safe range ($h_{p}\geq 0.5$ mm), the output charge decreases with increasing $h_{p}$ and gradually levels off, as the rising bending stiffness and the neutral-plane shift progressively suppress the in-plane bending strain of the wafer (the initial rising branch occurs only below the 0.5 mm safety baseline and is therefore outside the plotted range). Simultaneously, an increase in *a* expands the effective bending area, strongly amplifying the strain and charge output (scaling as $a^{4}$ in the analytical model). It should be emphasized that the selection of a=2.0 mm is directly governed by the precision machining limits of the alumina ceramic gasket. Within the available manufacturable dimensions, the maximum possible radius was selected to optimize the output, which is consistent with the theoretical trends observed in Figure 3c,d. Based on this parametric analysis, balancing the mechanical safety, manufacturing constraints, and the necessity to maximize piezoelectric output, the optimal internal structural parameters were determined as a=2.0 mm and $h_{p}=0.5$ mm (as explicitly marked by the solid symbols in the response curves).

Finally, to validate this specific optimal design and physically justify the 10 MPa full-scale measurement range, a mechanical safety verification was conducted on the finalized structure ($h_{m}=0.2$ mm, $h_{p}=0.5$ mm, a=2.0 mm). FEA results demonstrated that under the 10 MPa full-scale pressure, the maximum principal stress within the core sensing element (PZT-5H wafer) peaked at 73.8 MPa at the central suspended region, as shown in the stress distribution contour map (Figure 3e). While the intrinsic flexural strength of PZT-5H is 114.8 MPa [28], this 73.8 MPa peak stress closely approaches but safely remains below the conservative 80 MPa engineering safety limit provided by the PZT-5H wafer supplier for this component. This stress evaluation supports the mechanical safety of the brittle PZT-5H wafer under the defined 10 MPa full-scale condition, providing a physical basis for defining 10 MPa as the maximum full-scale range. Furthermore, Figure 3f confirms the linear relationship between the output charge and the applied pressure for this final optimized design, yielding a converged simulated sensitivity of approximately 4.96 pC/kPa (mesh-independent finest mesh; see Table 1).

## 4. Experimental System and Test Design

**Figure 4:**
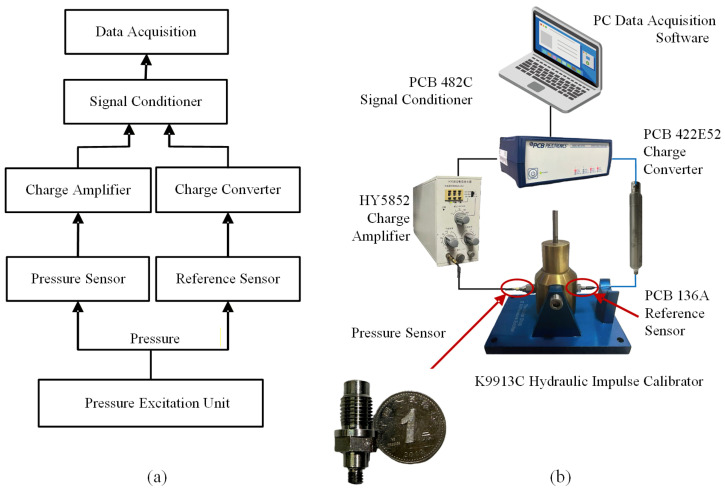
Schematic diagrams of the sensor quasi-static test system: (**a**) Principle schematic diagram and (**b**) actual test system composed of test equipment.

As shown in Figure 4a, the quasi-static test system comprises seven components: a pressure excitation unit, the sensor under test, a reference sensor, a charge amplifier, a charge converter, a signal conditioner, and a data acquisition module. The excitation unit applies the same pressure simultaneously to the sensor under test and the reference sensor; the resulting charge signals are converted to voltages by the charge amplifier and charge converter, then collected through the signal conditioner and data acquisition module. The actual test system is shown in Figure 4b. To resolve the sensor’s charge output accurately, the system uses low-noise, high signal-to-noise-ratio equipment throughout.

The pressure excitation unit adopts a PCB Model K9913C hydraulic pulse calibrator (PCB Piezotronics, Inc., Depew, NY, USA). This device operates based on the hydrodynamic pulse principle, realizing pressure loading by precisely adjusting the drop hammer mass and drop height, with a pressure rise time of approximately 3ms. The loading process is stable and controllable, with a pressure maintenance stability error of ≤±0.2% FS. Strictly speaking, the calibrator is an impulse-type device; the loading is treated here as quasi-static because, at each pressure level, the sensor output and the reference-sensor reading reach a common quasi-equilibrium plateau that is sampled synchronously, so that the static pressure-charge characteristic can be extracted independently of the loading rate. The sensor under test is installed in the built-in pressure-bearing test chamber of the device via a standard threaded interface, and the mounting surface is sealed with a metal gasket to prevent pressure leakage. The reference sensor employs a PCB 136A tourmaline hydraulic pressure calibration transfer standard, with a measurement range of 0–103.4 MPa (15 kpsi), a nominal sensitivity of 0.029 pC/kPa (±15%), and a non-linearity of ≤0.5% FS. It is coaxially installed with the sensor under test in the test chamber, real-time collecting the true value of the applied pressure for load calibration and loading stability verification. Weak charge signals output by the sensor under test are transmitted to a HY5852 charge amplifier (Jiangsu Lianergy Electronic Technology Co., Ltd., Yangzhou, China) via a shielded cable. This amplifier supports a wide range of charge-to-voltage conversion, with an input impedance of $\geq 10^{12}\;\Omega$, which can effectively match the high-impedance output characteristics of piezoelectric sensors, completing signal amplification and preliminary noise reduction. The gain setting of the HY5852 charge amplifier was calibrated and incorporated into the charge conversion, so that the reported output is expressed directly in terms of the collected charge (pC); its output scaling was set to 0.1 mV/Unit, with a high-pass (lower) cut-off frequency of 30 Hz and a low-pass (upper) cut-off frequency of 30 kHz. Because the pressure was applied as short (∼3 ms) pulses, the 30 Hz lower cut-off does not attenuate the peak charge response, which develops well before any significant charge decay. Charge signals output by the reference sensor are transmitted to a PCB 422E52 charge converter (PCB Piezotronics, Inc., Depew, NY, USA) through a shielded cable. The converted voltage signals and the amplified signals of the sensor under test are synchronously connected to a PCB 482C signal conditioner (PCB Piezotronics, Inc., Depew, NY, USA). This conditioner supports multi-channel synchronous signal processing, and filter parameters can be selected via internal jumpers to further suppress environmental noise. Processed signals are recorded by supporting data acquisition software in a computer. Prior to the experiments, an insulation test was performed using a Keithley 6517B electrometer (Keithley Instruments, Cleveland, OH, USA) to ensure that the insulation resistance between the sensor under test and the housing/test chamber is ≥100MΩ, so as to prevent leakage current from interfering with the accuracy of test signals. The insulation test was repeated after the completion of all quasi-static measurements, confirming that the insulation resistance remained above 100 MΩ.

The temperature-dependent performance was evaluated using a separate quasi-static pneumatic pressure system, since the operating temperature range of the PCB 136A reference sensor (−26 °C to +37 °C) does not cover the elevated-temperature regime. Pressure was supplied and regulated by a Mensor CPC4000 pneumatic pressure controller (Mensor, San Marcos, TX, USA), which provides a control stability better than 0.005% FS and an accuracy of up to 0.02% IS-50 (Mensor IntelliScale-50), ensuring an accurate and stable pressure reference at high temperatures. The sensor under test was heated in a muffle furnace, while the reference sensor was kept outside the heated zone and was therefore not exposed to high temperature. Measurements were taken at ten temperature set points from 25 °C to 250 °C in 25 °C increments (25, 50, 75, 100, 125, 150, 175, 200, 225, and 250 °C). At each set point, the sensor was held for 30 min to allow the sensing element and fixture to reach thermal equilibrium before a constant pressure of 1 MPa was applied, and the resulting charge output was acquired through a Kistler Type 5015 charge amplifier (Kistler Instrumente AG, Winterthur, Switzerland). Five repeated heating runs were performed to verify the consistency of the temperature response; cooling-cycle (thermal hysteresis) measurements were not conducted in this study and are left for future work. The insulation resistance was verified to remain ≥100MΩ at room temperature before and after the elevated-temperature runs; a dedicated measurement of insulation resistance at each high-temperature set point was not performed and is identified as a limitation to be addressed in future work.

Dynamic performance evaluation was carried out at the Beijing Institute of Aerospace Metrology and Measurement Technology (Beijing, China) on a shock-tube dynamic pressure calibration facility, which generates steep step pressure signals that simulate transient pressure impacts under actual operating conditions. The JBG-01 shock tube produces a reflected step pressure (calibrated reflected-step range 0.01–7 MPa) with a rise time of ≤0.1 μs and a platform (plateau) pressure duration of 4–17 ms; because this excitation rise time is more than two orders of magnitude shorter than the observed sensor response, the input can be regarded as a near-ideal step. The transient charge response was conditioned by a Kistler Type 5018 charge amplifier (maximum input charge $1.0\times 10^{5}$ pC) and recorded by a GEN3i high-speed transient data-acquisition system (HBK, Darmstadt, Germany) at a sampling rate of 25 MHz (40 ns sampling interval; ±25 V input range), while Kistler 603CAB reference pressure sensors (0–10 MPa) monitored the applied step amplitude. The ≤0.1 μs excitation rise time and the 25 MHz acquisition rate jointly ensure that neither the excitation nor the measurement chain limits the observed sensor response, so that a microsecond-scale leading edge can be faithfully resolved. By analyzing the sensor’s response to these step signals, its leading-edge response time and dominant resonance can be evaluated. All acquired signals were processed, analyzed, and plotted using MATLAB R2025b (MathWorks, Natick, MA, USA) and OriginPro 2024 (OriginLab, Northampton, MA, USA).

## 5. Results and Discussion

### 5.1. Quasi-Static Test Results and Discussion

**Figure 5:**
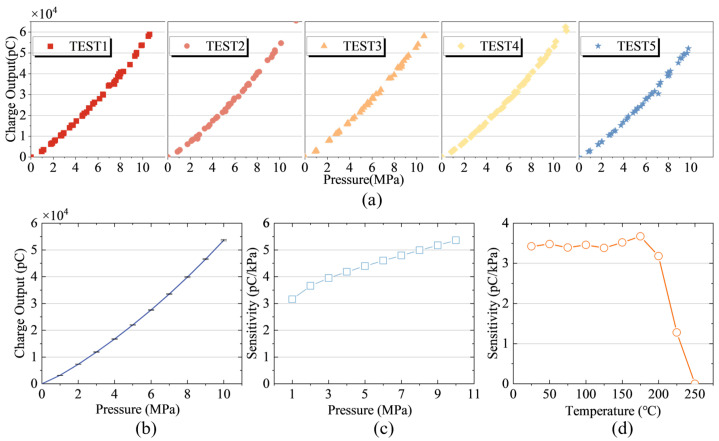
Quasi-static test results of the sensor (five repeated tests; charge amplifier: HY5852, 30 Hz–30 kHz bandwidth): (**a**) raw data of five tests, (**b**) fitted pressure-output charge curve with error bars using a second-order polynomial calibration, $Q=191.42\;p^{2}+3482.76\;p-272.14$ ($R^{2}=0.99995$), where *Q* is in pC and *p* is in MPa, (**c**) variation trend of secant sensitivity with pressure, and (**d**) relationship between sensitivity and operating temperature (five heating runs at 1 MPa constant pressure).

In quasi-static testing, because sensors with the same design were damaged during range testing exceeding 10 MPa, the measurement range of the sensor was defined as 0 to 10 MPa, which covers the operating pressures of many medium-pressure fluid-power and thermal-fluid systems. Figure 5a displays the raw data from five quasi-static tests within this range. The fitted pressure-output charge curve in Figure 5b is well described by a second-order polynomial, $Q=191.42\;p^{2}+3482.76\;p-272.14$ (with *Q* in pC and *p* in MPa), yielding a coefficient of determination of $R^{2}=0.99995$. The sensor therefore behaves as a nonlinear (quadratic-response) device rather than a linear one. Accordingly, the device is not evaluated against a best-fit straight line, and a conventional linearity (nonlinearity) error is not an appropriate figure of merit here; instead, the deviation of the measured data from the above quadratic calibration is reported as the residual error. This nonlinearity is primarily attributed to the large-deflection mechanical deformation of the metal diaphragm and piezoelectric wafer under high pressure. Unlike traditional bulk piezoelectric sensors that typically exhibit lower sensitivity but maintain good linearity, this design employs a peripherally clamped suspended membrane structure. As the pressure approaches 10 MPa, the increased central deflection of the thin plate triggers a stress stiffening effect, leading to a nonlinear enhancement of the in-plane tension, which is the physical origin of the observed quadratic dependence of the output charge on pressure. Because this response is highly deterministic and repeatable, the quadratic calibration equation establishes a well-defined charge–pressure mapping, so that the applied pressure can be reliably recovered from the charge output despite the nonlinearity. The error bars in the figure represent the standard deviation of multiple measurements at each pressure point, reflecting the good stability of the output signal. Based on the five repeated tests, the type-A standard uncertainty of the output charge at each pressure point, evaluated as the standard deviation normalized to the full-scale output, is less than 0.5% FS, indicating good measurement reproducibility. The remaining measurement uncertainty mainly arises from the reference sensor, pressure loading stability, charge-amplifier conversion, and data-acquisition resolution.

Influenced by the aforementioned mechanical mechanism, the secant sensitivity of the sensor, defined as the ratio of the mean output charge to the applied pressure (S=Q/p), increases monotonically within the 0 to 10 MPa range, rising from approximately 3.16 pC/kPa at 1 MPa to 5.36 pC/kPa at 10 MPa as shown in Figure 5c. The overall fluctuation is controlled within 2.2 pC/kPa. The secant values in Figure 5c are computed from the measured mean charge at each pressure level rather than from the global quadratic fit; the two agree within the sub-1% FS fitting residual, with the difference being relatively larger at low pressure, where the residual is divided by a small pressure value. This load-dependent variation of sensitivity is a direct manifestation of the quadratic response. Accepting a deliberate trade-off of strict linearity in exchange for a substantial enhancement of the overall sensing response, compared with traditional cavity-free devices, constitutes the core design choice of this composite structure.

The finite element-predicted sensitivity of approximately 4.96 pC/kPa (Section 3) lies within this experimentally measured range and is closest to the values at the upper end of the pressure range. The deviation from the load-dependent experimental sensitivity is attributed to the idealized contacted interfaces and to the small simulated deflections of the stiff 0.2 mm diaphragm, for which the geometric stress-stiffening captured by the (geometrically nonlinear) FEA remains weak over 0–10 MPa; the larger stiffening observed experimentally is additionally influenced by interface compliance, contact, and preload effects not represented in the idealized model.

Furthermore, the temperature performance of the sensor was tested by applying a constant pressure of 1 MPa at different operating temperatures. The results in Figure 5d demonstrate that the sensor operates stably over a temperature range of 25 °C to 150 °C, where the output deviation at 150 °C relative to the room temperature of 25 °C remains within 3%, indicating good thermal stability within this range. Beyond 150 °C, however, the measured sensitivity decreases markedly and approaches zero near 250 °C. This behavior is consistent with the thermal depolarization of PZT-5H as the temperature approaches its Curie point ($T_{C}\approx 193$ °C) [29]: as $T\to T_{C}$, the remnant polarization and the piezoelectric coefficient $d_{31}$ degrade rapidly, leading to a pronounced loss of charge output. The stable operating window of 25–150 °C therefore corresponds to the temperature region safely below $T_{C}$. The stability within this window also confirms the good match in thermal expansion coefficients between the alumina ceramic and the piezoelectric wafer, which helps maintain sensitivity stability in fluctuating thermal environments below the Curie point.

**Figure 6:**
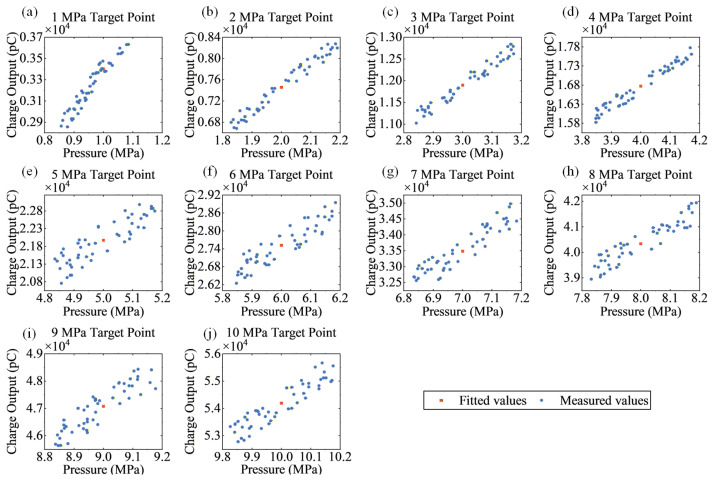
Measurement accuracy of the piezoelectric pressure sensor evaluated at different target pressure levels. (**a**–**j**) show the test results of 50 pressure points randomly selected near target pressures ranging from 1 MPa to 10 MPa, respectively.

Figure 6 presents the measurement accuracy of the sensor evaluated at various pressure points. Because the hydraulic pulse calibrator cannot accurately reproduce an identical pressure value on repeated loading, the conventional same-input repeatability could not be measured directly. Instead, at each target pressure level, 50 actual measurement points were collected within a range of ±0.2 MPa around the target, each yielding a pair of measured pressure $p_{i}$ and measured charge $Q_{i}$. For every point, the residual was computed as the difference between the measured charge $Q_{i}$ and the value $Q_{\mathrm{fit}}\left(p_{i}\right)$ predicted by the fitted calibration equation. The maximum error relative to the fitted calibration curve was then defined as the largest absolute residual divided by the full-scale output (53,616 pC at 10 MPa). It is evident that the measured points are closely distributed around the fitted calibration curve, and the resulting maximum error relative to the fitted curve is less than 1% FS. This low error indicates that, after calibration, the applied pressure can be recovered from the charge output with good accuracy across the full range, and that the interfacial contact and mechanical clamping structure maintained stable mechanical coupling under repeated high-pressure loading. It should be noted that loading–unloading hysteresis could not be evaluated with the present impulse-type calibrator, which applies discrete pressure pulses rather than continuous ramp cycles; this characterization is deferred to future work with a deadweight tester or other monotonic-loading apparatus.

### 5.2. Dynamic Test Results and Discussion

**Figure 7:**
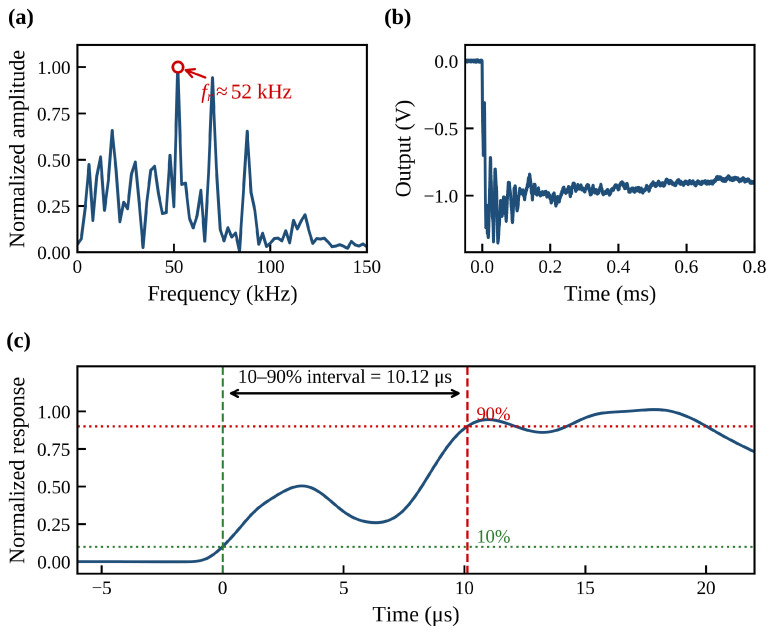
Dynamic performance of the sensor evaluated via shock-tube step-response testing under a reflected step of ∼0.5 MPa (sampling rate: 25 MHz; charge amplifier: Kistler 5018, DC mode, low-pass filter disabled): (**a**) amplitude spectrum of the post-step transient, obtained with a Hanning window after a zero-phase 8 kHz high-pass filter that removes the slowly varying baseline, showing a dominant resonance at ∼52 kHz; (**b**) raw recorded time-domain step-response waveform, showing the rapid leading edge and the subsequent damped ring-down; (**c**) enlarged view of the leading edge, from which the 10–90% leading-edge interval was determined as 10.12 μs. Panels (**a**,**c**) are computed from the baseline-subtracted, sign-normalized waveform, whereas panel (**b**) shows the raw recorded voltage.

The resonant frequency was extracted from the damped ring-down of the step response rather than from a Fast Fourier Transform (FFT) of the entire record. A step signal concentrates most of its energy at the lowest frequencies, and the FFT bin spacing equals the reciprocal of the record length; consequently, a full-record FFT places its apparent maximum among the lowest discrete spectral lines, which originate from the finite record duration, the step component, and the slow recovery trend, together with the associated spectral leakage, rather than from a mechanical mode. Instead, an FFT (Hanning window) was applied to a 0.5 ms transient window following the step, after removing the slowly varying baseline with a zero-phase (forward–backward) fourth-order Butterworth high-pass filter; its 8 kHz cut-off lies far below the ∼50 kHz ring-down, so it only suppresses the slow baseline drift and does not participate in locating the high-frequency peak. The dominant peak was identified at approximately 52 kHz (Figure 7a) and cross-checked against a damped-sinusoid fit, $A\;e^{-\zeta \omega_{n}t}\cos\left(\omega_{d}t+\varphi \right)$, applied to the leading cycles of the ring-down, which gives a natural frequency $f_{n}\approx 46$ kHz and a damping ratio ζ≈0.34. The two estimates are of the same order and together indicate a dominant ring-down mode of approximately 50 kHz. This resonance represents the response of the sensing element together with its packaging, mounting condition, and shock-tube excitation path, rather than the isolated material resonance of the PZT-5H wafer alone.

The time-domain performance is illustrated in Figure 7b, with an enlarged view of the leading edge in Figure 7c. The 10–90% leading-edge interval, defined as the interval for the signal to rise from 10% to 90% of its first main peak, was read directly from the 25 MHz waveform as 10.12 μs; because the 40 ns sampling interval is more than two orders of magnitude shorter than this value, the sampling resolution does not limit the reading. This leading edge corresponds to the onset of the damped resonant response described above: its duration is close to one half of the resonant period (∼10 μs at 50 kHz), confirming that the leading-edge interval and the resonance are consistent descriptions of the same underdamped second-order response. By contrast, the rise time-equivalent bandwidth $0.35/t_{r}$, in which $t_{r}$ denotes the measured 10–90% leading-edge interval of 10.12 μs, does not represent the flat-amplitude measurement bandwidth. For amplitude-accurate measurement, the usable bandwidth of an underdamped sensor is commonly estimated to be about one-fifth of its resonant frequency [30], corresponding to approximately 10 kHz in the present mounted configuration. This estimate suggests suitability for medium- and low-frequency pressure-pulsation monitoring, although a dedicated swept-frequency calibration is still required to determine the amplitude-flat measurement bandwidth more rigorously.

The measurement chain does not limit these results. The JBG-01 shock tube delivered a reflected step of approximately 0.5 MPa (monitored by the 603CAB reference sensors) with a rise time of ≤0.1 μs, more than two orders of magnitude faster than the sensor response; the Kistler 5018 charge amplifier provided a −3 dB frequency range of ≈0–200 kHz with the low-pass filter disabled (group delay ∼2 μs); and the transient recorder sampled at 25 MHz. Combining the amplifier’s ∼1.75 μs rise time in quadrature with the measured value indicates an instrument contribution below 2%, so the recorded edge reflects the response of the sensor in its mounted configuration. The small local fluctuation on the rising edge in Figure 7c is attributed to the non-ideal shock front and transient wave reflections within the shock-tube fixture and mounting cavity, rather than to abnormal sensor behavior. Within this interpretation, the sensor tracks transient pressure changes with good fidelity, making it well suited to applications that demand a high signal-to-noise ratio and good transient tracking but not an ultra-high-frequency bandwidth. Particular applications include dynamic pressure-pulsation monitoring of hydraulic pumps, valves, and actuators in fluid-power systems, as well as water-hammer and pressure-wave monitoring in thermal and power-plant fluid systems where the 150 °C operating capability is advantageous.

### 5.3. Comparison with Representative Piezoelectric Pressure Sensors

The quasi-static and dynamic characterizations in Section 5.1 and Section 5.2 establish the key performance metrics of the proposed sensor: a charge sensitivity of 3.16–5.36 pC/kPa over a 0–10 MPa range, stable operation up to 150 °C, and a fast transient response (∼50 kHz dominant resonance and a 10.12 μs leading edge). To assess these metrics against the current state of the art, Table 3 compares the proposed sensor with representative recent piezoelectric pressure sensors reported in the literature, along with mature commercial dynamic pressure transducers, in terms of material, pressure range, sensitivity, operating temperature, resonant frequency, rise time, repeatability, accuracy and application.

It should be noted that the sensitivities in Table 3 retain the units reported by each source: voltage-mode sensors are rated in mV/kPa, which depends on the built-in amplifier gain, whereas this work and the charge-mode devices are rated in pC/kPa, reflecting the intrinsic charge output independently of the amplifier; the two conventions are not directly interconvertible. Among the directly comparable charge-mode devices, a high charge sensitivity has so far been reported only over narrow, kPa-level ranges or, in the MPa regime, at substantially reduced output, whereas the proposed sensor sustains 3.16–5.36 pC/kPa across the full 0–10 MPa range. Together with its stable operation up to 150 °C, this simultaneous combination of high charge output and wide measurement range distinguishes the proposed sensor from the charge-mode designs listed in Table 3.

## 6. Conclusions

This study developed a composite layered piezoelectric pressure sensor based on a 316L stainless-steel diaphragm, a thickness-poled PZT-5H wafer, and a perforated alumina ceramic gasket. The centrally suspended PZT-5H region supported by the annular alumina gasket promotes bending deformation of the piezoelectric wafer and enhances charge generation under pressure loading. A laminated composite plate model and finite element analysis were used to guide the structural design and to evaluate the effects of key geometric parameters on stress distribution and output charge.

The optimized sensor operated over a pressure range of 0–10 MPa. The pressure-output response was well fitted by a second-order polynomial, indicating a nonlinear but calibratable response. The pressure-dependent secant sensitivity increased from approximately 3.16 pC/kPa at 1 MPa to 5.36 pC/kPa at 10 MPa, while the maximum error relative to the fitted calibration curve was less than 1% FS. Temperature testing showed that the output deviation at 150 °C relative to room temperature remained within 3%, indicating stable operation within the 25–150 °C operating window.

Dynamic shock-tube testing showed a resonance on the order of 50 kHz, extracted from the damped ring-down of the step response, together with a measured 10–90% leading-edge interval of 10.12 μs. These two metrics are mutually consistent—the latter being close to one half of the resonant period—and characterize the response of the sensor in its mounted configuration rather than the isolated material resonance of the sensing element. These results suggest that the combination of high charge sensitivity, fast transient response, and stable operation up to 150 °C makes the proposed sensor suitable for dynamic pressure-pulsation monitoring in fluid-power systems and for water-hammer and pressure-wave monitoring in thermal and power-plant fluid systems.

Future work will focus on reducing pressure-dependent nonlinearity, separating the intrinsic sensor response from shock-tube and packaging effects, and conducting more comprehensive reliability tests, including loading–unloading hysteresis, thermal cycling, insulation stability, and fatigue under repeated dynamic loading. Replacing PZT-5H with higher-coefficient active materials, such as relaxor-ferroelectric single crystals (e.g., PMN-PT) or emerging lead-free ceramics (e.g., KNN-based systems), is also a promising route to further raising the charge output of the present structure. This gain, however, must be weighed against practical constraints: many relaxor single crystals have comparatively low Curie temperatures that would conflict with the 150 °C operating window targeted here, and both single crystals and lead-free ceramics typically present greater machining brittleness and higher cost. PZT-5H was therefore retained in this work as a balanced compromise between piezoelectric activity, thermal stability, manufacturability, and cost; the structural optimization strategy proposed here is nonetheless material-agnostic and can be transferred to these alternative materials when their thermal and processing limitations are acceptable for a given application.

## Figures and Tables

**Table 1:** Mesh independence study: element-size settings, full-scale (10 MPa) output charge, and corresponding full-scale sensitivity for three successively refined meshes.

Mesh (Calibration)	Elements	Max. Size (mm)	Min. Size (mm)	Charge @10 MPa (pC)	Sensitivity (pC/kPa)
Coarse (Fine)	12,115	0.80	0.100	52,067	5.21
Fine (Finer)	35,437	0.55	0.040	48,913	4.89
Finest (Extra fine)	100,361	0.35	0.015	49,629	4.96

The mesh labels in parentheses denote the predefined COMSOL element-size calibrations. The relative deviations of the full-scale output charge with respect to the preceding (coarser) mesh are 6.1% (Coarse-to-Fine) and 1.4% (Fine-to-Finest), indicating mesh convergence.

**Table 2:** Key physical and electromechanical properties of the materials used in the FEA simulation.

Property	316L Stainless Steel	Alumina Ceramic (99%)	PZT-5H
Density, ρ (kg/m^3^)	7980	3900	7500
Young’s Modulus, *E* (GPa)	193	380	60.6 ^*a*^
Poisson’s Ratio, ν	0.28	0.22	0.31 ^*a*^
Piezoelectric Constant, $d_{31}$ (pC/N)	/	/	−274
Piezoelectric Constant, $d_{33}$ (pC/N)	/	/	593
Relative Permittivity, $\epsilon_{33}^{T}/\epsilon_{0}$	/	/	3400

^*a*^ In the FEA, the PZT-5H wafer was defined by full anisotropic stiffness, piezoelectric coupling, and permittivity matrices from the COMSOL built-in material library. The Young’s modulus and Poisson’s ratio listed here are the equivalent in-plane (radial–circumferential) values derived from the corresponding compliance matrix and used in the analytical laminate model.

**Table 3:** Comparison of the proposed sensor with representative piezoelectric pressure sensors from the literature and commercial products.

Ref./Model	Piezoelectric Material	Range	Sensitivity	Operating Temp.	Resonant Freq./Rise Time	Accuracy	Target Application
Liang & Wang [19]	Flexible PZT thick-film	0–55 kPa	813 mV/kPa	−20 to 70 °C	6 kHz/—	Linear, R ≈ 0.99	Blast testing
Dzuba et al. [21]	AlGaN/GaN diaphragm (MEMS)	0–36 kPa	4.4 pC/kPa	—	—/—	Freq.-independent	Dynamic MEMS pressure
Dabrowski & Golonka [22]	PZT	0–6 MPa	0.71 pC/kPa	—	—/—	Linear (charge mode)	High-pressure/industrial
Li et al. [23]	PZT-5A	0–1.2 MPa	1.13 pC/kPa	—	237 kHz/—	<0.1% (lin. & rep.)	Blast/shock wave
PCB 113B24 [31]	Quartz	0–6.9 MPa	0.73 mV/kPa	−73 to 135 °C	≥500 kHz/≤1 μs	0.08% lin.; 0.03% rep.	Blast pressure
PCB 136A [32]	Tourmaline	0–103.4 MPa	0.029 pC/kPa	−26 to 37 °C	≥1 MHz/≤3 μs	≤0.5% FS	Calibration standard
This work	PZT-5H	0–10 MPa	3.16–5.36 pC/kPa	25–150 °C	∼50 kHz/10.12 μs	<1% FS	Fluid-power/thermal-fluid

—: not reported; FS: full scale.

## Data Availability

All data supporting reported results are included in the manuscript.
